# Potent inhibition of VEGFR-2 activation by tight binding of green tea epigallocatechin gallate and apple procyanidins to VEGF: Relevance to angiogenesis

**DOI:** 10.1002/mnfr.201400478

**Published:** 2015-01-22

**Authors:** Christina W A Moyle, Ana B Cerezo, Mark S Winterbone, Wendy J Hollands, Yuri Alexeev, Paul W Needs, Paul A Kroon

**Affiliations:** Institute of Food Research, Norwich Research ParkNorwich, UK

**Keywords:** Angiogenesis, Dietary polyphenols, EGCG, Nitric oxide synthase, Procyanidin

## Abstract

**Scope:**

Excessive concentrations of vascular endothelial growth factor (VEGF) drive angiogenesis and cause complications such as increased growth of tumours and atherosclerotic plaques. The aim of this study was to determine the molecular mechanism underlying the potent inhibition of VEGF signalling by polyphenols.

**Methods and results:**

We show that the polyphenols epigallocatechin gallate from green tea and procyanidin oligomers from apples potently inhibit VEGF-induced VEGF receptor-2 (VEGFR-2) signalling in human umbilical vein endothelial cells by directly interacting with VEGF. The polyphenol-induced inhibition of VEGF-induced VEGFR-2 activation occurred at nanomolar polyphenol concentrations and followed bi-phasic inhibition kinetics. VEGF activity could not be recovered by dialysing VEGF-polyphenol complexes. Exposure of VEGF to epigallocatechin gallate or procyanidin oligomers strongly inhibited subsequent binding of VEGF to human umbilical vein endothelial cells expressing VEGFR-2. Remarkably, even though VEGFR-2 signalling was completely inhibited at 1 μM concentrations of polyphenols, endothelial nitric oxide synthase was shown to still be activated via the PI3K/Akt signalling pathway which is downstream of VEGFR-2.

**Conclusion:**

These data demonstrate for the first time that VEGF is a key molecular target for specific polyphenols found in tea, apples and cocoa which potently inhibit VEGF signalling and angiogenesis at physiological concentrations. These data provide a plausible mechanism which links bioactive compounds in food with their beneficial effects.

## 1. Introduction

Angiogenesis, the formation of new blood vessels from pre-existing ones, is known to play an important part in the development and destabilisation of atherosclerotic plaques [1–3]. Angiogenesis occurs when there is an imbalance between pro-angiogenic and anti-angiogenic growth factors and vascular endothelial growth factor (VEGF) is the most important pro-angiogenic growth factor in humans [4–6]. VEGF stimulates cellular responses by binding to type III receptor tyrosine kinases on the cell surface which causes the receptors to dimerise and become activated through transphosphorylation. VEGF receptor-2 (VEGFR-2) is the major mediator of the mitogenic, angiogenic and permeability enhancing effects of VEGF [6,7].

Data from numerous epidemiological studies have indicated that individuals who consume the largest quantities of fruits and vegetables in their diets have lower rates of mortality and morbidity for a range of chronic diseases such as cancer and cardiovascular disease [8]. Polyphenols present in fruits and vegetables have been implicated as playing a significant role in the protective effects of these foods [9]. Green tea catechins [10], red wine polyphenolic compounds [11], certain anthocyanins [12], grape seed extracts [13] and cinnamon extract procyanidins [14] have been reported to inhibit VEGF-induced angiogenesis. Using human whole genome microarrays, we have shown that the main effects of treating resting and cytokine-stimulated human umbilical vein endothelial cells (HUVECs) with an apple procyanidin (tannin) fraction were changes in angiogenic functions and the underlying signalling pathways [15]. These observations were particularly interesting because whereas relatively low concentrations of the apple procyanidin polyphenol fraction induced significant changes in the expression of many hundreds of genes, treatments with epicatechin or a procyanidin dimer did not cause any significant changes in gene expression under the same conditions.

While a number of dietary polyphenolic compounds have been shown to inhibit VEGF-induced VEGFR-2 phosphorylation [16], the underlying molecular mechanisms are not known. Polyphenols may inhibit VEGF-induced VEGFR-2 phosphorylation in three ways: (i) by binding directly to the VEGF molecule in a way that prevents VEGF from binding to its receptor; (ii) by binding to the VEGF receptor in a way that prevents VEGF from binding and/or activating the receptor; or (iii) by interacting with intracellular components involved in phosphorylation of VEGFR-2. The original aim of the present study was to determine whether polyphenols interact with the VEGF ligand or with components of the endothelial cells (VEGFR-2, intracellular kinases, etc.) to inhibit VEGFR-2 activation. We present data which show that epigallocatechin gallate (EGCG) and procyanidin oligomers inhibit VEGF-mediated VEGFR-2 phosphorylation by directly interacting with VEGF and reducing its binding to the receptor. We also report some interesting features of the binding between the polyphenols and VEGF and data on the downstream effects of polyphenol-mediated inhibition of VEGFR-2 activation.

## 2. Materials and methods

### 2.1. Cell culture

HUVECs were obtained from Lonza (Slough, UK), and maintained in endothelial cell growth medium-2 (Lonza). The cells were cultured at 37°C in an atmosphere at 5% CO_2_.

### 2.2. Preparation of the isolated tetrameric procyanidin fraction and EGCG

The procedure used to purify the apple procyanidin fraction with a degree of polymerisation of 4 (dp4) has been previously described (briefly detailed in Supporting Information) [17]. EGCG was isolated from green tea using methanolic extraction, followed by chromatographic separations using a pad of MN polyamide SC2 eluted batchwise followed by preparative reverse phase HPLC (×2) using a 250 × 41.4 mm id Dynamax-60A, 83-241-C (8 μm, 60 Å) C-18 column. Appropriate fractions containing pure EGCG (as determined by LC-MS) were combined, evaporated and stored at −20°C before use. A detailed description of the EGCG isolation process is provided in the Supporting Information.

### 2.3. Polyphenol treatment of HUVECs

Confluent HUVECs were washed two times with warm PBS before addition of either vehicle control (≤ 0.1% DMSO), VEGF (human recombinant VEGF_165_, R&D Systems, Abingdon, UK) or a mixture of VEGF and polyphenol (dp4 or EGCG). Treatments were for various times and concentrations and were performed using endothelial basal medium (endothelial cell growth medium-2 with no serum or growth factors). After treatments, cells were lysed with radio-immunoprecipitation assay buffer containing protease and phosphatase inhibitors. Protein content of lysates was determined by a bicinchoninic acid assay (Sigma, Poole, UK).

### 2.4. Phosphorylated VEGFR-2 ELISA

Levels of phosphorylated VEGFR-2 in lysates were measured using a PathScan Phospho-VEGFR-2 (Tyr1175) sandwich ELISA kit (Cell Signalling Technology, Hitchin, UK) following the manufacturer's instructions.

### 2.5. Western blot analysis for VEGR-2, VEGF, AKT, phospholipase C gamma 1 (PLCγ1) and endothelial nitric oxide synthase (eNOS)

Western blot analysis was performed using antibodies directed against phospho-VEGFR-2 (Tyr 1175), VEGFR-2, phospho-AKT (Ser 473), AKT, phospho-PLCγ1 (Tyr783), PLCγ1, phospho-eNOS (Ser 1177) and eNOS (Cell Signalling Technology) and goat-anti-VEGF and anti-goat IgG-HRP antibodies (R&D Systems) following the manufactures’ instructions. Details can be found in the Supporting Information.

### 2.6. Dialysis of VEGF-polyphenol complexes

Slide-A-Lyzer MINI Dialysis Unit (Thermo Scientific, Hitchin, UK) with a molecular weight cut off of 3500 Da was used for dialysis assay. VEGF (900 ng/mL) was incubated with or without the polyphenol (36 μM) in 20 mM ammonium bicarbonate buffer pH 7.0 for 30 min at room temperature. Then, samples were added to the units and subsequently placed in a flotation device and dialysed against 1 L of the buffer at 4°C, with the buffer replaced after 2 and 24 h. Post-dialysis, the retentates were carefully recovered using a micropipette and diluted 36-fold with basal medium to give a final concentration of 25 ng/mL VEGF and 1 μM of polyphenol which was used to treat HUVECs. At the same time, VEGF alone and VEGF with polyphenol at the same concentrations (900 ng/mL and 36 μM, respectively) were incubated at 4°C for 2 and 24 h without dialysis, then diluted to a final concentration of 25 ng/mL VEGF and 1 μM of polyphenol and used to treat HUVEC as controls.

### 2.7. Prediction of polyphenol-binding sites on VEGF

The crystal structure of VEGF was obtained from the protein data bank (PDB code: 2vfp) [18]. 2vpf was prepared for docking (removal of water molecules, addition of polar hydrogens and gasteiger charges) and a PDB, partial charge (Q) and atom type (T) file required for docking was prepared using AutoDock Tools (ADT) v1.5.4 [19]. The 3D structure of EGCG was obtained from the PubChem chemical library (compound ID: 65064) [20], dp4 was drawn in Accelrys Draw 4.0 and the structure energy minimised. Non-polar hydrogens were merged, gasteiger charges added, rotatable bonds set and PDB, partial charge (Q) and atom type (T) files prepared with ADT for both EGCG and dp4.

Docking of EGCG or dp4 into 2vpf was performed using AutoDock Vina v1.1.0 [21]. The docking area was defined by a box centred on 2vpf which included the whole protein. Docking results were ranked according to binding free energy. The structure with the lowest free binding energy was chosen for the optimum docking conformation. Residues in 2vpf in which may potentially interact with the docked polyphenols were identified with ADT.

### 2.8. Binding of VEGF to HUVECs

HUVECs were removed from flasks by incubation with accutase (PAA laboratories, Yeovil, UK) for 5 min at 37°C, cells were washed with PBS and resuspended in PBS + 0.1% foetal calf *serum* + 0.02% sodium azide + 4 mM EDTA (PBSA-EDTA). One micrograms per liter Biotinylated recombinant human VEGF was mixed with 40 and 400 μM of EGCG or dp4 in PBS and incubated at room temperature for 5 min prior to addition to the HUVECs. The sensitivity of the flow cytometric detection of biotinylated VEGF required that a much higher concentration (1 μg/mL) was used than in the pVEGFR-2 inhibition assays conducted with HUVECs assessed by ELISA (25 ng/mL), and so the polyphenol concentration was also increased 40-fold so that the ratio of concentrations was maintained. To measure the effect of EGCG or dp4 on VEGF binding to VEGF receptors in HUVECs, we utilised a Fluorokine® Biotinylated Human VEGF kit (R&D systems) following the manufacturer's instructions. FITC intensity was measured with a Becton Dickinson FC500 flow cytometer (10 000 events were acquired), and data were analysed using WINMDI 2.9.

### 2.9. Microarray analysis and RT-PCR

Changes in gene expression in HUVECs in response to treatment with VEGF (10 ng/mL), an apple tetrameric procyanidin fraction (dp4; 1 μM) and VEGF pre-treated with the tetrameric procyanidin fraction were examined using Affymetrix GeneChip® Human Exon 1.0 ST Arrays with a vehicle-only control treatments included alongside. HUVECs were firstly pre-incubated with basal medium (5 min) and then subjected to the treatments for 6 h prior to RNA extraction (all treatments performed in triplicate, one well per replicate). RNA was extracted from HUVECs using the RNeasy® Mini Kit (Qiagen Ltd., UK) according to the manufacturer's protocol. The optional on-column DNase digestion was included. The quality of RNA was assessed by the Nottingham Arabidopsis Stock Centre (Nottingham, UK) using an RNA Nano LapChip kit and an Agilent 2100 Bianalyzer and the quality and quantity of RNA was also assessed using a Beckman DU-640 spectrometer.

The microarray data were analysed using the Bioconductor software [22] and the Aroma Affymetrix package [23]. Statistical analysis was performed using the Linear Model for MicroArrays (Benjamini and Hochberg adjusted *p*-values). The Database for Annotation, Visualization and Integrated Discovery v6.7 was used to identify Gene Ontology categories associated with specific gene lists [24].

The VEGF-induced up-regulation of angiopoietin-2 (ANGPT2) which is the gene encoding ANGPT2 (a protein that promotes angiogenesis induced by VEGF) in HUVECs was confirmed using qRT-PCR (TaqMan). Pre-designed gene assays for ANGPT2 was purchased from Applied Biosystems (Assay ID: Hs00169867_m1). Target gene mRNA levels were determined by real-time RT-PCR using the ABI Prism 7500 Sequence Detection System (Applied Biosystems) and normalised to the housekeeping gene 18S (Sigma). The real-time RT-PCR reactions were carried out in a Microamp Optical 96-well plate in a total volume of 20 μL per well containing TaqMan® RNA-to-C_T_™*1-Step* Kit, 20 ng total RNA and appropriate concentrations of primers and probes. Real-time RT-PCR conditions were as follows: one cycle of 48°C for 30 min, one cycle of 95°C for 10 min followed by 40 cycles at 95°C for 15 s and one cycle at 60°C for 1 min.

### 2.10. Statistical analysis

Statistical analyses were performed using Graphpad Prism software. Student's *t*-test was used to test significant differences between samples.

## 3. Results

### 3.1. VEGFR-2-activating activity of VEGF in the presence of EGCG or dp4

**Figure 1:**
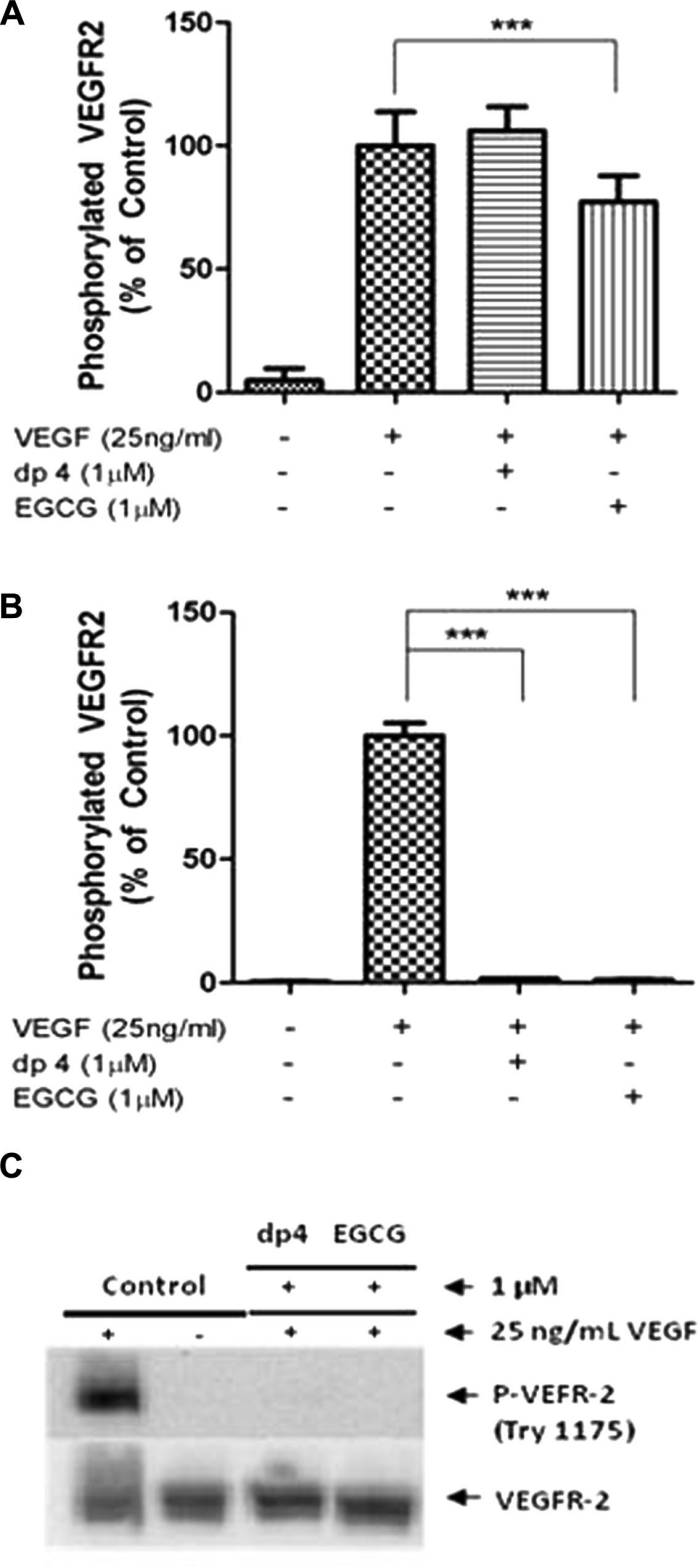
Apple dp4 and EGCG inhibit VEGF-induced VEGFR-2 phosphorylation by interacting with the VEGF molecule. HUVECs were exposed (A) to 1 μM apple dp4 or EGCG for 4 h before removal of the polyphenol (washing with PBS) and the addition and incubation of 25 ng/mL VEGF for 5 min, or (B and C) for 5 min to 1 μM apple dp4 or EGCG previously incubated with 25 ng/mL VEGF for 5 min. Phosphorylated VEGFR-2 was determined by ELISA (A and B) and Western blot (C). ***p < 0.001 compared to the stimulated cells. Data are expressed as mean ± SD (*n* = 6 (A and B)). (C) Densitometric analysis of *n* = 3 Western blots.

First, we incubated HUVECs with the polyphenols (1 μM dp4 or EGCG) or vehicle control for 4 h before rinsing the cells (twice with PBS) and changing the media to one containing only VEGF (25 ng/mL) for 5 min. Phosphorylated VEGFR-2 was barely detectable in vehicle-treated HUVECs but VEGF treatment resulted in substantial and significant increases in pVEGFR-2 (Fig. 1A). The dp4 cell treatment was completely ineffective in inhibiting VEGF-induced pVEGFR-2 (Fig. 1A). This demonstrates that either dp4 is not interacting with VEGFR-2 or any sub-cellular kinase or the interaction is weak because any putative inhibitory effect was completely lost after a simple washing step. Pre-treatment of HUVECs with EGCG before washing and subsequent addition of VEGF caused a 26% reduction in pVEGFR-2 compared to treatments with VEGF alone. This observation shows that at 1 μM EGCG after 4 h can interact with components of the HUVEC cells to moderately reduce VEGF-induced VEGFR-2 activation.

The second experimental design involved pre-mixing the VEGF (25 ng/mL) and polyphenol (1 μM EGCG or dp4) for only 5 min prior to treating the HUVECs with the mixture for 5 min. Remarkably, both polyphenols caused complete inhibition of VEGF-induced VEGFR-2 activation (Fig. 1B) without affecting VEGFR-2 total protein (Fig. 1C). These data support the notion that both polyphenols interact directly with the VEGF protein, and that the result of this interaction is complete inhibition of the VEGFR-2-activating capacity of VEGF. The data also demonstrate that at 1 μM concentrations of the two polyphenols, direct interaction with VEGF is the only effective mechanism resulting in inhibition of VEGF-induced VEGF activation by dp4 and is the dominant mechanism for EGCG.

### 3.2. Nature of the interaction between the VEGF protein and the polyphenols

**Figure 2:**
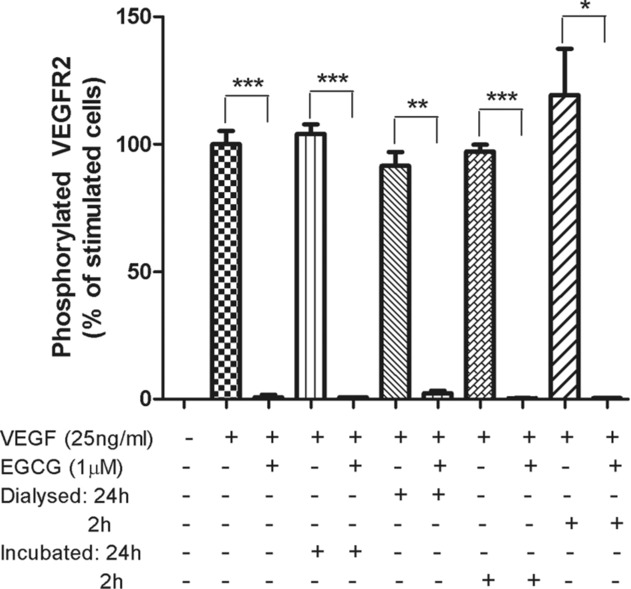
Inhibition of VEGFR-2 activation activity of VEGF by EGCG is retained after removal of unbound polyphenol using a dialysis membrane. HUVECs were treated for 5 min with pre-incubated basal medium containing 25 ng/mL VEGF, 25 ng/mL VEGF and 1 μM EGCG, or samples prepared by dialysis (including the controls). For the dialysed samples, VEGF was incubated with or without EGCG for 30 min at room temperature prior to dialysis for 2 or 24 h at 4°C. The controls were incubated in the same conditions without dialysis. The retentate was diluted in medium to give a final concentration of 25 ng/mL VEGF and 1 μM EGCG. Phosphorylated VEGFR-2 was determined by ELISA. *p < 0.05, **p < 0.01, ***p < 0.001 compared to their stimulated cells, respectively. Data expressed as mean ± SD (*n* = 3).

Having demonstrated that EGCG and dp4 interact directly with the VEGF protein, we explored the nature of this interaction further by investigating whether it was the result of weak or strong interactions and whether these were covalent or non-covalent binding. First, we dialysed VEGF-polyphenol complexes and determined whether or not VEGF recovered its ability to activate VEGFR-2. Our data show that untreated VEGF caused strong activation of VEGFR-2 in HUVEC after dialysis (Fig. 2) whereas EGCG-treated VEGF did not exhibit any VEGFR-2 phosphorylation activity post-dialysis. Similarly, dialysed dp4-treated VEGF was unable to phosphorylate VEGFR-2 in HUVECs (data not shown). These observations likely indicate that the EGCG and dp4 bind tightly to VEGF and are not released after an extended period (24 h) of dialysis. This is consistent with a covalent or strong non-covalent interaction, but not with an easily reversible non-covalent interaction. Second, EGCG- and dp4-treated VEGF samples were subjected to various forms of gel electrophoresis. Using SDS-PAGE, polyphenol-treated VEGF exhibited exactly the same migration properties under both reducing and non-reducing conditions as un-treated VEGF (Supporting Information Fig. 1). This strongly suggests that covalently linked VEGF-polyphenol adduct(s) were not formed and that binding between VEGF and the two polyphenols was the result of (strong) non-covalent binding. We also attempted to determine if there were differences in the p*I* of polyphenol-treated VEGF and un-treated VEGF but the high intrinsic p*I* of VEGF (p*I* = 8.0–8.5) [25] precluded us from visualising VEGF or putative VEGF adducts on the gels, presumably because the VEGF migrated off the top (cathodic) edge of the gels (data not shown). We also analysed VEGF and polyphenol-treated VEGF using MALDI-TOF MS to try and detect changes in the mass of VEGF after polyphenol treatment. However, the mass spectra obtained for VEGF and polyphenol-treated VEGF were very similar and no modifications were observed (data not shown). The lack of observed increases in the mass of VEGF post-treatment with the polyphenols is not consistent with the formation of covalent bonds between the VEGF and the polyphenols, and it likely indicates that the non-covalent binding involved in complex formation is disrupted during ionisation such that only masses corresponding with free VEGF are observed in the MALDI-TOF analysis. The combination of results obtained from SDS-PAGE and MALDI-TOF MS do not support the notion that the binding between VEGF and polyphenol is due to a covalent interaction. Bearing in mind that VEGF activity was not recovered from polyphenol-treated VEGF samples following dialysis, it is most likely that the polyphenol-mediated inhibition of VEGF is the result of strong non-covalent interactions between VEGF and the polyphenols.

### 3.3. Kinetics of inhibition of VEGF activity by EGCG and dp4

**Figure 3:**
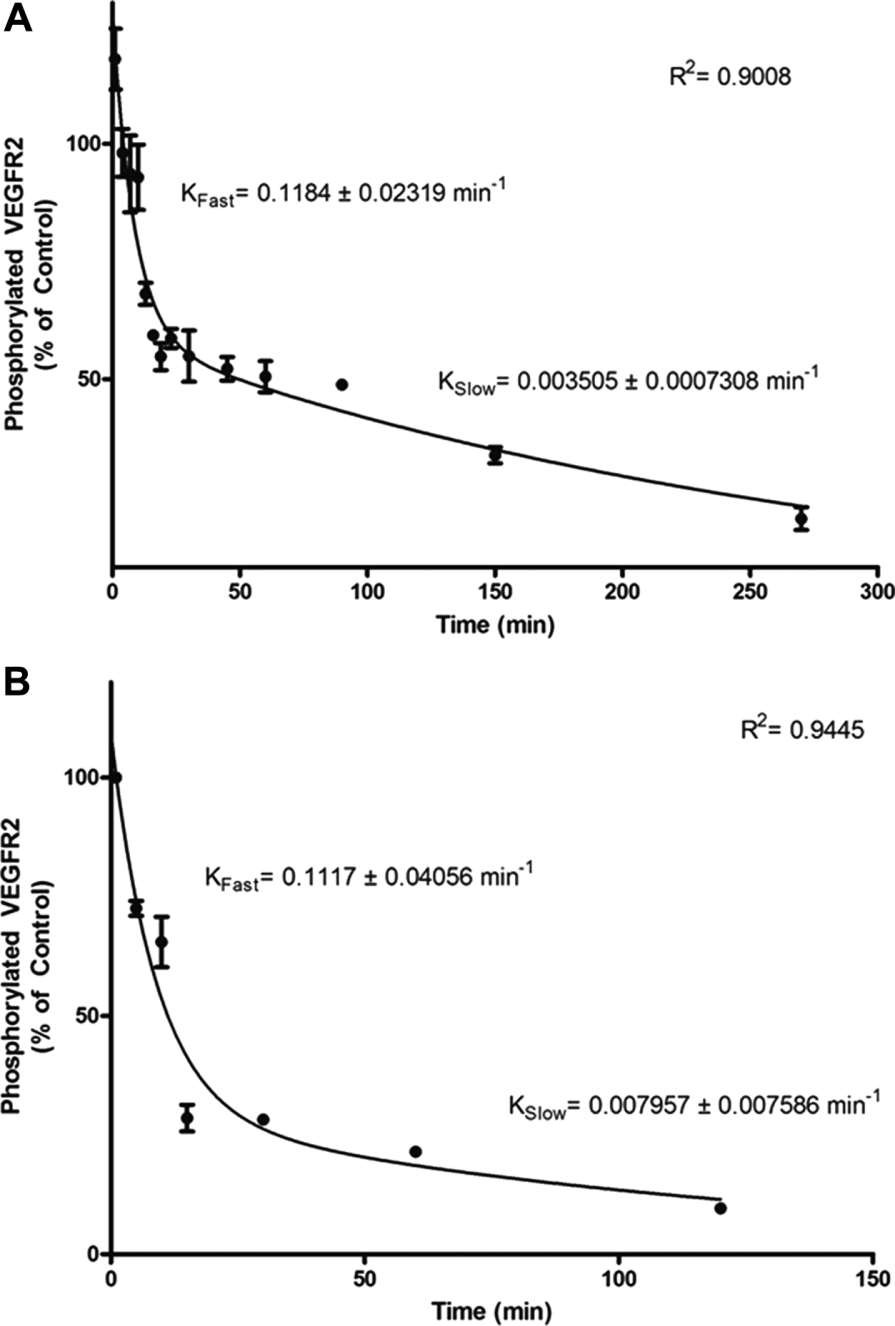
EGCG and apple dp4 inhibit VEGF-induced VEGFR-2 phosphorylation in a time-dependent manner. VEGF (25 ng/mL) and 62.5 nM EGCG (A) or 200 nM apple dp4 (B) were pre-incubated in basal medium for 1, 4, 7, 10, 13, 16, 19, 23, 30, 45, 60, 90, 150 and 270 min (A) or 5, 10, 15, 30, 60 and 120 min (B). HUVECs were then treated for 5 min with the pre-incubated VEGF and EGCG or apple dp4 for the times indicated. The cells were lysed and the amount of phosphorylated VEGFR-2 was quantified by ELISA. Data are expressed as mean ± SD (*n* = 2).

In order to investigate the kinetics of VEGF inhibition by the polyphenols, VEGF was incubated with EGCG (62.5 nM) or dp4 (200 nM) for extended periods of time (1–270 min and 1–120 min, respectively) after which the VEGFR-2-activating activity of VEGF was determined by treating HUVECs with the polyphenol-treated VEGF (5 min). The data clearly shows that EGCG-mediated inhibition of VEGF activity is time dependent (Fig. 3A), which is not consistent with the very rapid (<1 s) time scales over which classic freely reversible enzyme-substrate complex equilibria are formed. Further, the EGCG data fitted very well to a two-phase model (two-phase decay) in which an initial fast exponential decay phase (*K*_Fast_ = 0.1184 ± 0.02 319 min^−1^) was followed by a slow exponential decay phase (*K*_Slow_ = 0.003505 ± 0.0007308 min^−1^). Similar results were obtained with dp4 but with different rates (Fig. 3B).

### 3.4. In-silico studies of the binding of EGCG and dp4 to VEGF

**Figure 4:**
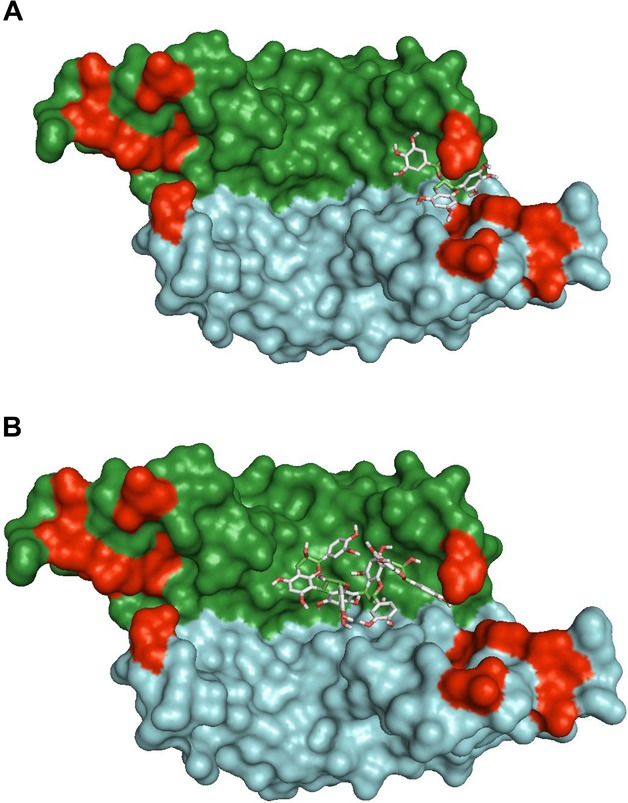
Computed highest affinity binding sites for EGCG (A) and dp4 (B) to VEGF; using AutoDock Vina v1.1.0 software. Surface representation of VEGF dimer with the individual monomers coloured in green and blue. Amino acid residues identified as important in VEGFR-2 binding (Ref 38) are coloured in red.

EGCG was predicted to bind into a groove at the pole of VEGF (Fig. 4A) with a binding affinity of −8.1 kcal/mol. dp4 was predicted to bind to a region of VEGF that is adjacent to the groove that EGCG is predicted to occupy (Fig. 4B), with an affinity of −8.2 kcal/mol. We also identified potential residues on VEGF that EGCG or dp4 may interact with based on the predicted most energetically favourable binding sites (Table 1). EGCG was predicted to interact with 13 residues on both subunits of VEGF and form hydrogen bonds with three residues (ASP34, LYS48 and SER50). dp4 was predicted to interact with 15 residues of VEGF including five residues via hydrogen bonds (SER50, ASN62, ASP63, GLU64, GLU67, CYS68).

**Table 1:** Residues on 2vpf predicted to interact with docked EGCG or dp4

EGCG		dp4	
Protein chain		Protein chain	
A	B	A	B
GLY59	ASP34a)	GLN37	SER50a)
CYS60	PHE36	GLU38	CYS51
CYS61	ILE46	ARG56	
ASN62	LYS48a)	CYS57	
ASP63	SER50a)	GLY58	
GLU64		GLY59	
GLU67		CYS61	
CYS68		ASN62a)	
		ASP63a)	
		GLU64a)	
		GLU67a)	
		CYS68a)	
		HIS99	

a) Indicate predicted hydrogen bonds.

### 3.5. Effect of polyphenols on VEGF binding to endothelial cells

**Figure 5:**
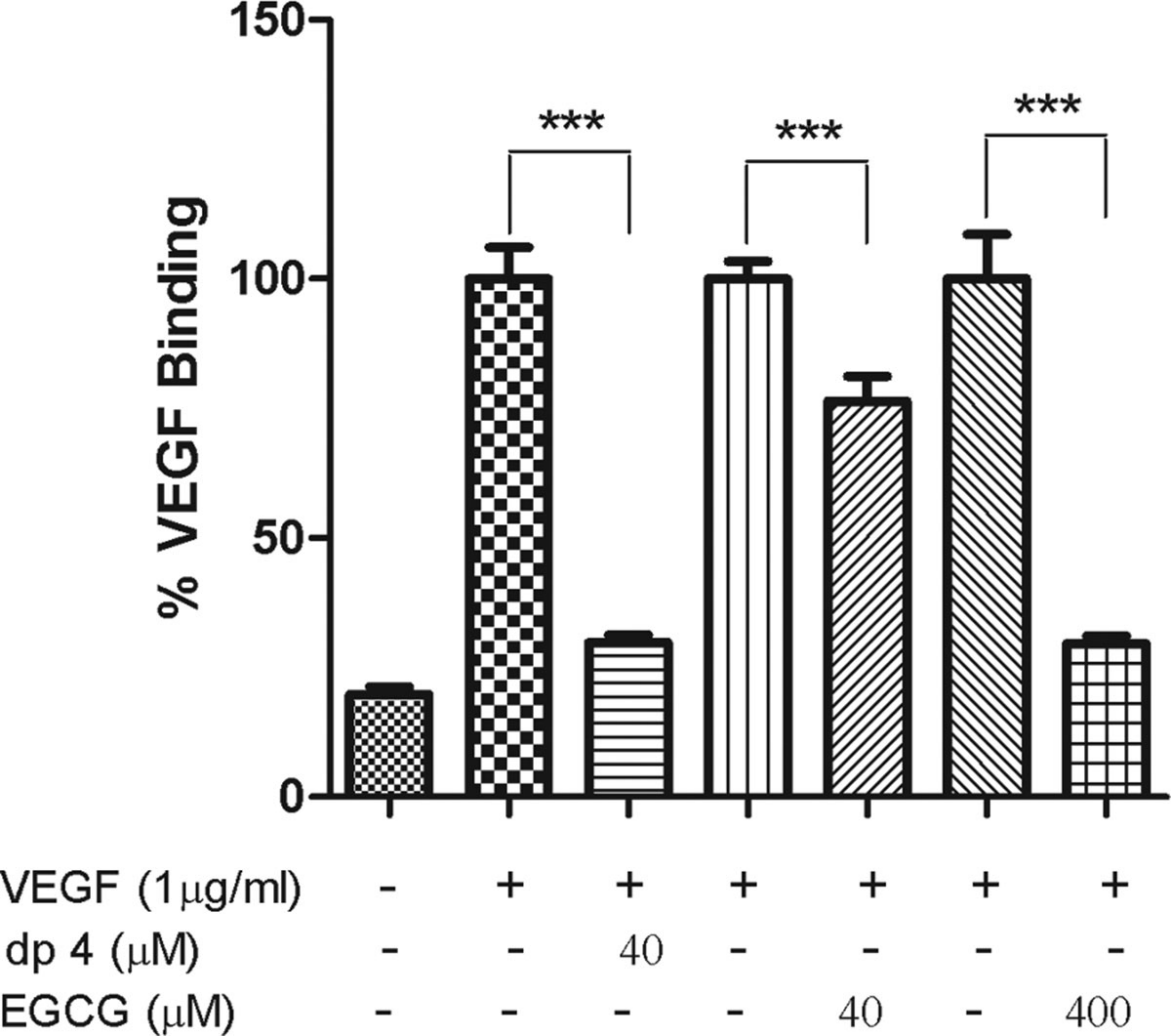
EGCG and apple dp4 reduced binding of VEGF to endothelial cells. HUVECs in single-cell suspension in PBSA-EDTA were treated with preincubated 40 μM apple dp4 or 40 and 400 μM EGCG and 1 μg/mL biotinylated VEGF for 30 min at 4°C. Then avidin-FITC was added and the cells were incubated for a further 30 min at 4°C in the dark. Bars represent means ± SD expressed as a percentage of FITC intensity after normalisation. ***p < 0.001 compared to their respective controls. Data are expressed as mean ± SD (*n* = 8).

The inhibitory effects of the polyphenols on the VEGFR-2 activity of VEGF may, or may not be, a consequence of the polyphenol preventing binding of VEGF to VEGFR-2 on the endothelial cells. We explored the effects of the polyphenol treatments of VEGF on its ability to bind to the surface of HUVECs. Using flow cytometry, we show that dp4 treatment almost completely inhibited binding of biotinylated VEGF to HUVEC (*p* < 0.001) at the same molar ratio as used in the previous experiments (25 ng/mL:1 μM, VEGF:polyphenol; Fig. 5). Treatment with EGCG at 40 μM also significantly reduced binding of VEGF to HUVECs (*p* < 0.001), but only by 20%, but at 400 μM EGCG binding was inhibited by >90% (*p* < 0.001; Fig. 5).

### 3.6. Downstream effects of polyphenol-induced inhibition of VEGF signalling on HUVECs

**Figure 6:**
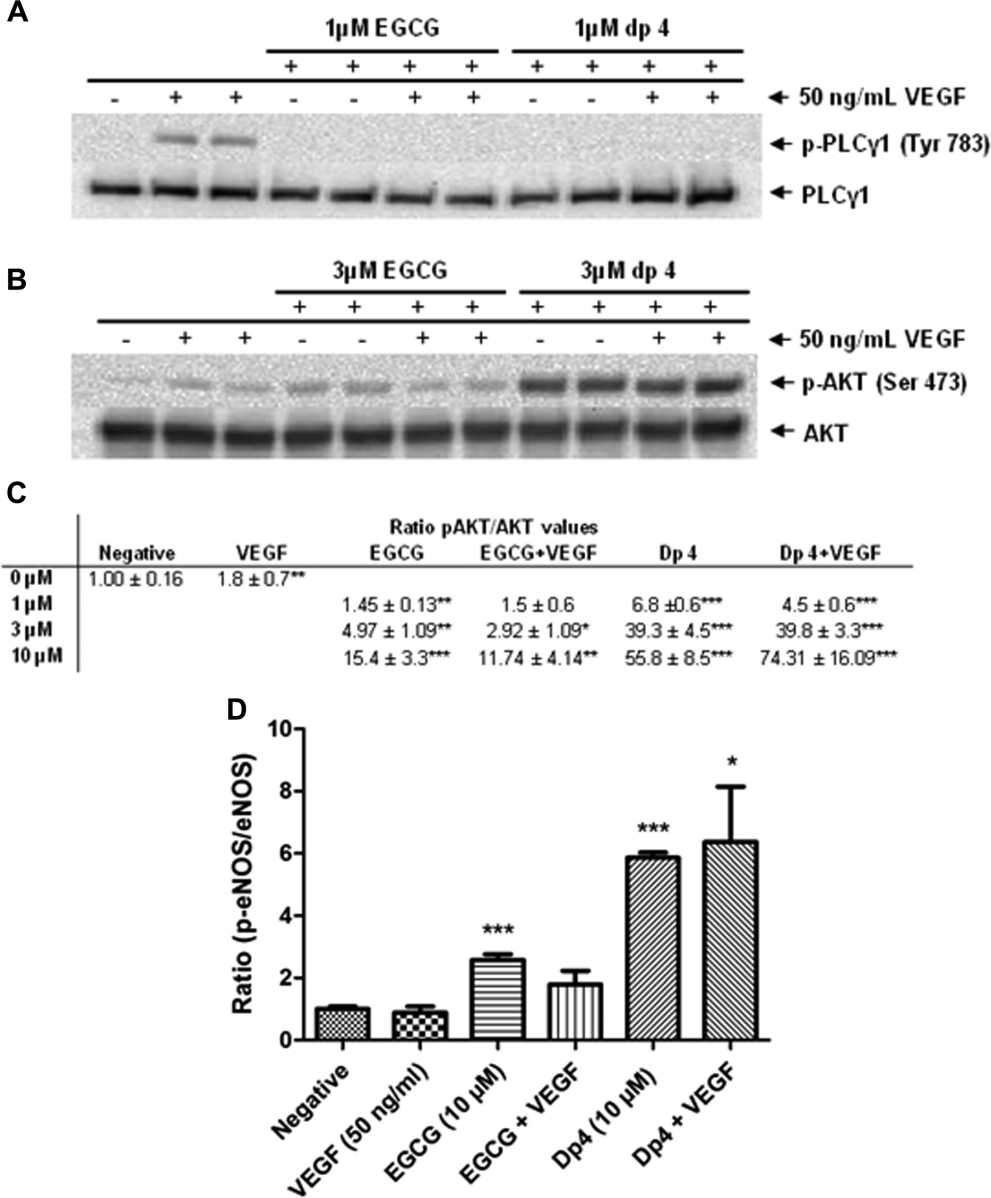
Apple dp4 and EGCG inhibits phosphorylation of PLCγ1 and activates phosphorylation of AKT in a dose-dependent manner and activate phosphorylation of eNOS. HUVECs were treated with pre-incubated (5 min) basal medium containing 50 ng/mL VEGF and 1 μM apple dp4 or EGCG for 10 min (A); 1, 3 or 10 μM dp4 or EGCG for 60 min (B and D) (just shown 3 and 10 μM data, respectively). (C) Representation of the ratio for pAKT/AKT at different polyphenol concentrations is shown. Cells were lysed and the proteins were separated on an SDS-PAGE gel and probed for the presence of the corresponding antibodies. *p < 0.05; **p < 0.01; ***p < 0.001 versus negative control. Densitometric analysis of *n* = 3 Western blots.

First, we checked that inhibition of the VEGFR-2-activating activity of VEGF by polyphenols also prevented signalling events downstream of pVEGFR-2. Our data show that PLCγ1, one of the first signalling pathway proteins phosphorylated in response to VEGFR-2 activation, was phosphorylated in HUVECs treated with VEGF, but VEGF-induced activation of PLCγ1 was completely blocked by dp4 and EGCG at 1 μM without affecting PLCγ1 total protein (Fig. 6A). These data confirm that dp4 and EGCG-mediated inhibition of VEGF-induced activation of VEGFR-2 also prevents downstream signalling through PLCγ1. Akt (activated later in the VEGF signalling cascade but also by several other signalling pathways) was weakly phosphorylated when treated with VEGF, but VEGF-induced phosphorylation of Akt was not inhibited by treatment with either EGCG or dp4. In fact, phosphorylation of Akt was induced by EGCG and dp4, both in the presence and absence of VEGF (Fig. 6B). Subsequently, it was shown that both polyphenols caused dose-dependent increases in phosphorylated Akt and the pAKt/Akt ratio, both in the presence and absence of VEGF, and that dp4 was a stronger Akt activator than EGCG (Fig. 6C). In light of our observation that EGCG and dp4 treatments caused activation of Akt, and since pAkt is known to activate the eNOS enzyme (peNOS), we assessed the effects of the polyphenol treatments on eNOS activation. Our data show that VEGF alone (50 ng/mL) did not affect the peNOS/eNOS ratio. In contrast, EGCG and dp4 alone both caused significant increases in the peNOS/eNOS ratio when applied at 10 μM (Fig. 6D) and also at 1 μM (data not shown). In the presence of VEGF, both EGCG and dp4 treatments caused increases in peNOS, but the effect was only significant for the dp4 treatment.

Finally, we used a non-targeted technique to examine the broader effects of polyphenol-mediated inhibition of VEGF activity in HUVECs. Comparing total RNA samples extracted from HUVECs treated with VEGF alone, dp4 alone, dp4-treated VEGF or vehicle-only control, we were able to show that dp4 completely abrogated the wide-ranging effects of the potent cytokine VEGF on HUVECs. VEGF alone (10 ng/mL) induced significant changes in the expression of multiple HUVEC genes (*p* < 0.05, *n* = 890; *p* < 0.01, *n* = 337; *p* < 0.001, *n* = 132) compared to the vehicle-only treated control. The significantly altered genes included A2M, ANGPT2, EGR3, PLAU, THBD, IL8, KLF2 and VCAM1 which have all been previously reported to be induced by VEGF treatment [26,27]. The VEGF-induced up-regulation of ANGPT2 HUVECs was confirmed using qRT-PCR (TaqMan), which showed a significant increase of 1.91-fold (*p* < 0.0001) compared with the control, which is very similar to the result obtained using microarrays (one increase of 1.81-fold, *p* < 0.001). Treatment with dp4 alone also caused significant changes in gene expression (1 μM; *p* < 0.05, *n* = 2689; *p* < 0.01, *n* = 446, *p* < 0.001, *n* = 94). Although some of the changes in transcripts caused by VEGF treatment alone and dp4 treatment alone were common (*n* = 75, *p* < 0.01), the majority were different (*n* = 262 unique for VEGF and *n* = 371 unique for dp4; all *p* < 0.01). But, the outstanding observation was that when the transcript profiles obtained from dp4-treated HUVECs were compared with the transcript profiles of HUVECs exposed to a mixture of dp4 and VEGF, there were no significant differences (Table 2). These data show that treatment of VEGF with dp4 completely blocks all the changes in gene expression usually induced by VEGF (132 changes *p* < 0.001, 337 changes *p* < 0.01, 890 changes *p* < 0.05).

**Table 2:** Identification of differentially expressed genes from pairwise comparisons

Comparison	*p* < 0.05	*p* < 0.01	*p* < 0.001
1. VEGF versus Negctrl	890 (350, 540)	337 (162, 175)	132 (74, 58)
2. dp4 versus Negctrl	2689 (1195, 1494)	446 (186, 260)	94 (37, 57)
3. VEGF and dp4 versus Negctrl	11 575 (7268, 4307)	6825 (4168, 2657)	511 (180, 331)
4. dp4 versus VEGF	1031 (454, 577)	524 (206, 318)	235 (83, 152)
5. VEGF and dp4 versus VEGF	3426 (1675, 1751)	1159 (370, 789)	360 (95, 265)
6. VEGF and dp4 versus dp4	0	0	0

The up-regulated and down-regulated genes are shown in brackets with the up-regulated gene preceding the down-regulated genes (i.e. up-regulated, down-regulated).

dp4 = 1 μM apple procyanidin fraction dp4; Negctrl = control (vehicle DMSO); VEGF = 10 ng/mL VEGF.

## 4. Discussion

In the present study, we have shown that the polyphenols EGCG from green tea and procyanidin oligomers from apples potently inhibit VEGF-induced VEGFR-2 signalling and subsequent angiogenesis at concentrations which may be achieved through diet. The inhibition was the result of a direct interaction of the polyphenol with the VEGF peptide which is a relatively slow process resulting in completely inactivated VEGF with reduced VEGFR-2-binding capacity. These findings provide scientific evidence to support the notion that VEGF is a molecular target for specific polyphenols such as EGCG and procyanidin oligomers and that direct binding of polyphenols to VEGF is the likely mechanism of inhibition at physiological polyphenol concentrations.

The ability of EGCG and procyanidins including tetramers to inhibit VEGF-mediated angiogenesis is well known and has been widely reported by various authors [14,10,28–30]. Additionally, numerous reports have provided evidence that certain polyphenols are able to inhibit VEGF-induced VEGFR-2 phosphorylation [13,14] but the mechanisms of inhibition were not directly investigated. Some researchers have proposed that polyphenols do not inhibit VEGFR-2 signalling by interacting with the VEGF receptor because there was no reduction in VEGFR-2 expression following polyphenol treatment [30], while other researchers stated that polyphenols bind to VEGFR-2 and thus prevent VEGF from binding to its receptor [10,12]. These two theories were based on pre-treatment of the cells with a high polyphenol concentration (10–25 μM) for an extended period of time (18–24 h) with subsequent removal of the media, and treatment of cells with VEGF. In view of the fact that polyphenol concentrations very rarely exceed 1 μM in blood after a polyphenol-rich meal, the data presented in this report demonstrate that the mechanism described here for the first time, namely direct interaction between the polyphenol and VEGF which renders the VEGF completely inactive, is more likely to occur in vivo. We have shown that this process can occur at <100 nM for EGCG and at 200 nM for procyanidin dp4 (Fig. 3). We have also shown that direct treatment of HUVECs with EGCG (1 μM) but not dp4 for 4 h caused a modest reduction (26%) in subsequent VEGF-induced VEGFR-2 activation (Fig. 1A). This shows that EGCG can interact directly with HUVECs to inhibit VEGFR-2 activation, and this may be via a similar mechanism to that described by Weber et al. [31] who reported that EGCG (50 μM) reduced the receptor-binding capacity of platelet-derived growth factor (PDGF) and showed that EGCG was incorporated into different cell compartments including cell surface membranes after extended incubation periods (4–24 h). But, at physiological concentrations of polyphenol (typically <1 μM in blood plasma), the most likely mechanism by which EGCG and dp4 inhibit VEGF signalling is by directly interacting with VEGF and blocking its ability to activate the receptor.

Although we have reported the results of some preliminary investigations into the interaction between the polyphenols and VEGF, the nature of the interaction remains somewhat unclear. Data presented here show that exposing VEGF to EGCG or dp4 completely inhibits the VEGFR-2-activating capacity of the VEGF, and that VEGFR-2-activating activity cannot be restored by dialysing the polyphenol-treated VEGF (Fig. 2). These observations strongly suggest that the polyphenols bind directly to VEGF in a way that prevents it from binding to and/or activating its receptor, and suggest that binding is either covalent or strong non-covalent. In contrast, we were not able to observe increases in the molecular mass of VEGF after treatment with either EGCG or dp4, using either MALDI-TOF MS or SDS-PAGE under reducing conditions. Although it is feasible that a covalently linked polyphenol-VEGF complex is highly unstable during MALDI-TOF MS analysis and only masses corresponding to deconjugated VEGF were observed, the SDS-PAGE results strongly suggest that covalent bonds are not involved. Further, a chemical route by which a procyanidin readily reacts with and covalently modifies a protein like VEGF is not apparent. The specific nature of the interaction between the polyphenols and the VEGF protein deserves attention in future research efforts in this research field.

The inhibition of VEGF activity occurs over extended periods of time when the concentration of polyphenols is in the physiological range (<1 μM; Fig. 3). This is important because following consumption of a polyphenol-containing meal, the concentrations of the polyphenols in blood will rise to a peak (usually within 1–6 h), and then decline back to or near baseline levels (usually within 12–24 h) [32]. The fastest rate of inhibition of VEGF will occur at the time of maximum polyphenol concentration in blood (= *T*_max_), but rather than observing a decline after this point (i.e. increases in VEGF activity as the polyphenol concentration decreases), it would be expected that VEGF activity would continue to decline due to the irreversible nature of the inhibition (Fig. 2), albeit with inhibition occurring at a slower rate.

Our observations of two phases in the inhibition kinetics are consistent with there being at least two distinct molecular events during the inhibition process. One possibility is that the VEGF protein undergoes a conformational change after binding of the polyphenol to the protein. This could involve a more rapid first phase in which a polyphenol molecule binds relatively weakly to VEGF and partly inhibits its activity, followed by a slower phase in which the VEGF changes conformation and as a result the strength of the binding to the polyphenol is increased dramatically (see Fig. 3). Polyphenols have been reported to induce conformational change in proteins. The binding of green tea polyphenols to casein proteins resulted in a conformational change of the secondary structure which led to the unfolding of the protein [33]. In contrast, the secondary structure alteration to β-lactoglobulin induced by green tea polyphenols resulted in stabilisation of the protein structure [34]. An alternative explanation of the two-phase inhibition curve is that there are two binding sites for the polyphenols, and the effect on VEGFR-2 activation of a first polyphenol molecule binding to the first site is greater than that for the binding of the second polyphenol molecule to the second site. Bearing in mind that VEGF functions as a dimer with the two monomers juxtapositioned in an antiparallel orientation, it is possible that the two binding sites are indeed the same on each VEGF molecule, and the in-silico modelling data reported here support this notion. Hence, it is possible that a VEGF dimer with a single-bound polyphenol molecule is partially inhibited, whereas VEGF that has polyphenol molecules bound to both the component monomers is completely inhibited.

The concentrations of green tea EGCG and apple procyanidin oligomers required to significantly inhibit VEGF activity are particularly noteworthy. For example, we have demonstrated complete inhibition of VEGF by 300 nM EGCG and 1000 nM dp4, and extensive inhibition of VEGF by EGCG and dp4 over 2–3 h time periods at very low concentrations (62.5 and 200 nM, respectively; Fig. 3). Although the concentration of procyanidins circulating in human plasma remains unknown, procyanidin trimer concentrations between 4 and 8.5 μM have been reported in rat plasma after the consumption of a high dose of procyanidins [35]. The reported concentrations of monomeric unconjugated catechins in plasma after the consumption of four cups of green tea are between 0.2 and 1 μM [30,36]. Thus, the inhibitory effect of dp4 and EGCG on VEGF observed in the present study occurs at concentrations that have been found in blood of human subjects or animal models after consuming dietary doses.

We used AutoDock Vina to examine potential binding sites for EGCG and dp4 on a crystal structure of VEGF deposited in the RCSB PDB Protein Data Bank. Although, the generated binding energies were relatively weak (approximately −8 kcal/mol) and not consistent with the tight binding that was observed experimentally, they are likely underestimated since VEGF is treated as a rigid structure and no account is taken for any conformational changes. The conformation of EGCG with the lowest binding affinity is located in a groove between the two monomers of VEGF by the VEGFR-2-binding site (Fig. 4A) formed by Asp63 and Glu64 on one side, Tyr36, Ile43 and Ile46 on the other side with Ser30 and Asp34 forming the bottom of the groove [37]. Ile46 and Glu64 are two of the three residues that contribute most to the binding energy of VEGF to VEGFR-2 [38]. dp4 binds to a region adjacent to the groove that EGCG occupies (Fig. 4B), which it is unable to access presumably due to steric hindrance. dp4 also interacts with Glu64 with which it is predicted to form a hydrogen bond. Additional residues identified as being part of the receptor-binding face include Phe36, Lys48, Asn62 and Asp63 [38] which may interact with EGCG or dp4. The VEGF homologue PDGF is able to bind VEGFR-1 but not VEGFR-2, out of the five most important residues identified by Muller et al. [38] for VEGFR-2 binding to VEGF only IL46 is not conserved in PDGF where it is changed to methionine, Lys48 and Asp 63 are also substituted further highlighting a role for these residues in VEGFR-2 binding. The docking studies show that EGCG and dp4 are predicted to bind VEGF close to or in a region associated with receptor binding where they can interact with residues implicated in VEGFR-2 binding.

Incubation of dp4 with labelled VEGF at the molar ratio which completely inhibited VEGFR-2 phosphorylation in HUVECs resulted in an almost total inhibition of binding of VEGF to HUVEC (Fig. 5). Incubation of the labelled VEGF with EGCG at the same molar ratio resulted in a reduction of 20%, probably due to VEGFs ability to bind other molecules present on cell surfaces such as neuropilins and heparin [39]. Further increasing the concentration of EGCG complete inhibited VEGF binding to the HUVECs. These results show that both dp4 and EGCG are able to prevent binding of VEGF to HUVECs and provides further evidence that the polyphenols inhibit VEGF-induced VEGFR-2 phosphorylation by interacting directly with the VEGF molecule and not receptors on the cell surface. Nevertheless, the relatively high concentration of VEGF required for the flow cytometry-based binding assays precluded us from testing the effects of polyphenol treatment of VEGF on VEGF binding to HUVECs at the lower concentrations used for the inhibition of VEGF-induced activation of VEGFR-2 assays. It is therefore possible that at the higher VEGF and polyphenol concentrations used, non-specific binding events occurred which might not occur at the lower concentrations.

Systematic reviews and meta-analyses of the anti-VEGF drugs bevacizumab, sorafenib and sunitinib have shown an increased risk of developing hypertension among users of these drugs [40,41]. VEGF stimulates the production of nitric oxide (NO) through the phosphorylation of AKT [30,42] and inhibition of VEGF signalling by anti-VEGF drugs would therefore be expected to decrease the production of NO [43]. On the other hand, data presented here have demonstrated that, in vitro at least, certain polyphenols inhibit VEGF signalling but still may induce NO bioavailability by increasing phosphorylation of AKT and also eNOS (Fig. 6). It is possible that polyphenols can effectively inhibit VEGF signalling at physiologically achievable concentrations but retain or even activate Akt and eNOS. Kim et al. [44] has already described a possible molecular mechanism mediated by intracellular signalling pathways requiring reactive oxygen species and Fyn that lead EGCG to activate phosphatidylinositol 3-kinase, Akt, eNOS and NO production in BAECs either by means of an unidentified specific cell surface receptor or by directly generating ROS in a receptor-independent fashion. A similar mechanism as described by Kim et al. [44] may explain the EGCG and dp4-induced activation of Akt and eNOS even when the polyphenols inhibit VEGF-induced VEGFR-2 activation.

In conclusion, we have identified VEGF as a key molecular target for certain polyphenols that are found in green tea and apples and demonstrated that binding and potent inhibition of VEGF takes place at polyphenol physiological concentrations achievable through diet.

## Abbreviations

ADT: AutoDock Tools
ANGPT2: angiopoietin-2
dp4: apple procyanidin oligomer degree of polymerisation 4
EGCG: epigallocatechin gallate
eNOS: endothelial nitric oxide synthase
HUVECs: human umbilical vein endothelial cells
NO: nitric oxide
PDB: protein data bank
PDGF: platelet-derived growth factor
PLCγ1: phospholipase C gamma 1
VEGF: vascular endothelial growth factor
VEGFR-2: VEGF receptor-2
