# Supplementary material for: Potent inhibition of VEGFR-2 activation by tight binding of green tea epigallocatechin gallate and apple procyanidins to VEGF: Relevance to angiogenesis
Source: Mol Nutr Food Res. 2015 Jan 22;59(3):401–12. doi: 10.1002/mnfr.201400478 (PMC4681316; doi:10.1002/mnfr.201400478)
Supplement: Supplementary file 1 — Figure 1 [file mnfr0059-0401-sd1.doc]

**Supporting information**

1. **Materials and Methods**

**2.2 Preparation of the isolated tetrameric procyanidin fraction and EGCG**

In brief, freeze-dried apple powder was extracted with 0.1% potassium disulfite and absorbed on a Sepabeads SP-850 (Sigma, Poole, UK) column. The polyphenol fraction was eluted with 50% ethanol and the polyphenol fraction was further absorbed onto a Diaion HP-20ss (Sigma, Poole, UK) column where any remaining un-wanted polyphenols (quercetin glycosides, chlorogenic acid and phloridzin) were removed. The procyanidin fraction was eluted with 25% ethanol and any impurities from the procyanidin fraction were removed by passing the fraction through a Biotage Snap FLASH Purification Cartridge HP-Sil (Biotage, Cardiff, UK) on a medium pressure liquid chromatography system. Finally, procyanidins were fractionated according to their dp by performing normal-phase chromatography using an Intersil PREP-Sil column (GL-Sciences Inc, Reading, UK).

Briefly, a 25% methanol (MeOH), 70°C extract of green tea was applied to a pad of MN polyamide SC2. The latter was washed with water, then eluted, stepwise, three times with 100% MeOH. Appropriate methanol fractions were pooled and evaporated, re-dissolved in 20% acetonitrile (MeCN), and subjected to preparative reverse phase HPLC on a 250 x 41.4 mm id Dynamax-60A, 83-241-C, 8 μm, 60 Å C-18 column. The column was eluted with a gradient of aq. 0.1% trifluoroacetic acid (TFA) and MeCN, with UV detection at 270 nm. Appropriate EGCG containing fractions- screened by analysis on an Agilent 1100 LC system equipped with a diode array UV detector and a G1956B SL single quadrupole, electrospray mass spectrometer (LC-MS) were combined, evaporated, re-dissolved in 20% MeCN and re-subjected to preparative hplc, now using a 0.1% aq. TFA-methanol gradient. Appropriate fractions containing pure EGCG, as determined by LC-MS were combined and evaporated, and stored at -20° before use.

**2.5 Western blot analysis for VEGR-2, VEGF, AKT, PLCγ1 and eNOS**

Protein lysates (30 μg) were mixed with NuPage LDS sample buffer (Invitrogen, Loughborough, UK), 50 mM DTT (Sigma, Poole, UK) and denatured by heating at 70ºC for 10 min. Proteins were then subjected to electrophoresis on NuPAGE 4-12% Bis-Tris gels (Invitrogen) before being transferred to 0.45 μM nitrocellulose membranes. Membranes were blocked with 5% BSA in TBST and probed overnight at 4ºC with antibodies directed against phospho-VEGFR-2 (Tyr 1175), VEGFR-2, phospho-AKT (Ser 473), AKT, phospho-PLCγ1 (Tyr783), PLCγ1, phospho-eNOS (Ser 1177) and eNOS (Cell Signalling Technology). Membranes were then incubated for 1h at room temperature with anti-rabbit IgG-HRP antibody (Cell signalling Technology), in 5% non-fat milk in TBST. Immunoreactive bands were detected using SuperSignal West Pico chemiluminescent substrate (Thermo Scientific, Hitchin, UK) and visualised on a Fluor-S MultiImager station (Bio-Rad, Hemel Hempstead, UK). Band densities were measured with Quantity One 1-D analysis software v4.5.2, and data are reported as the ratio of phosphorylated:total protein.

For detection of VEGF, 12% NuPAGE Bis-Tris Gels, goat-anti-VEGF and anti-goat IgG-HRP antibodies (R&D Systems, Abingdon, UK) were used. For non-reducing conditions DTT was omitted from and antioxidant was not added to running buffer.

1. **Results**


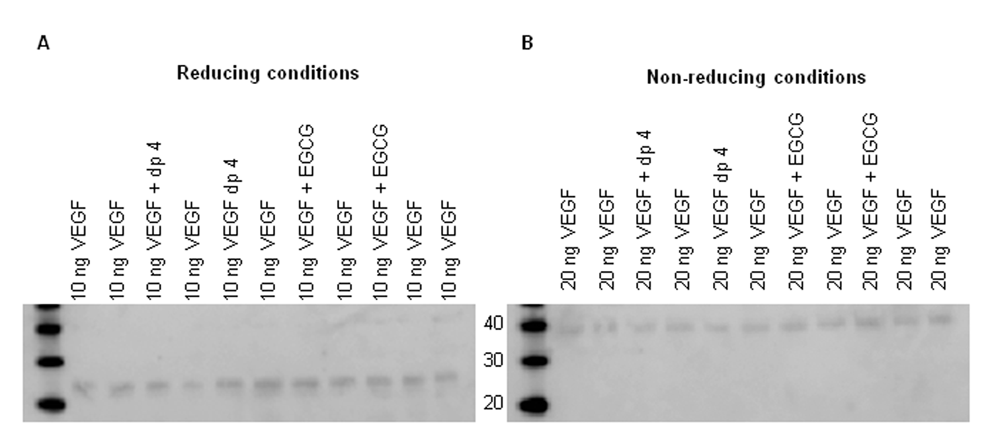


**Figure 1. Apple dp 4 and EGCG are not causing a modification of the VEGF protein mass under reducing or non-reducing conditions SDS-Page gels.** (A) VEGF (10 ng) was incubated for 5 min with or without 30.5 µM apple dp 4 or EGCG prior to reduction; or (B) VEGF (20 ng) was incubated with or without 61 µM apple procyanidin fraction dp 4 or EGCG for 5 min prior to the electrophoresis. (A and B) Densitometric nalysis of n=3 western blots
